# Treatment of a chemoresistant neuroblastoma cell line with the antimalarial ozonide OZ513

**DOI:** 10.1186/s12885-016-2872-2

**Published:** 2016-11-08

**Authors:** Don W. Coulter, Timothy R. McGuire, John G. Sharp, Erin M. McIntyre, Yuxiang Dong, Xiaofang Wang, Shawn Gray, Gracey R. Alexander, Nagendra K. Chatuverdi, Shantaram S. Joshi, Xiaoyu Chen, Jonathan L. Vennerstrom

**Affiliations:** 1 College of Medicine, Division of Pediatrics, University of Nebraska Medical Center, Omaha, NE USA; 2 Department of Pharmacy Practice and Pharmaceutical Sciences, University of Nebraska Medical Center, Omaha, NE USA; 3 Department of Genetics, Cell Biology and Anatomy, University of Nebraska Medical Center, Omaha, NE USA

**Keywords:** Neuroblastoma, Ozonide antimalarials, Metabolism, Cell cycle

## Abstract

**Background:**

Evaluate the anti-tumor activity of ozonide antimalarials using a chemoresistant neuroblastoma cell line, BE (2)-c.

**Methods:**

The activity of 12 ozonides, artemisinin, and two semisynthetic artemisinins were tested for activity against two neuroblastoma cell-lines (BE (2)-c and IMR-32) and the Ewing’s Sarcoma cell line A673 in an MTT viability assay. Time course data indicated that peak effect was seen 18 h after the start of treatment thus 18 h pre-treatment was used for all subsequent experiments. The most active ozonide (OZ513) was assessed in a propidium iodide cell cycle flow cytometry analysis which measured cell cycle transit and apoptosis. Metabolic effects of OZ513 in BE (2)-c cells was evaluated. Western blots for the apoptotic proteins cleaved capase-3 and cleaved PARP, the highly amplified oncogene MYCN, and the cell cycle regulator CyclinD1, were performed. These in-vitro experiments were followed by an in-vivo experiment in which NOD-scid gamma immunodeficient mice were injected subcutaneously with 1 × 10^6^ BE (2)-c cells followed by immediate treatment with 50–100 mg/kg/day doses of OZ513 administered IP three times per week out to 23 days after injection of tumor. Incidence of tumor development, time to tumor development, and rate of tumor growth were assessed in DMSO treated controls (*N* = 6), and OZ513 treated mice (*N* = 5).

**Results:**

It was confirmed that five commonly used chemotherapy drugs had no cytotoxic activity in BE (2)-c cells. Six of 12 ozonides tested were active in-vitro at concentrations achievable in vivo with OZ513 being most active (IC50 = 0.5 mcg/ml). OZ513 activity was confirmed in IMR-32 and A673 cells. The Ao peak on cell-cycle analysis was increased after treatment with OZ513 in a concentration dependent fashion which when coupled with results from western blot analysis which showed an increase in cleaved capase-3 and cleaved PARP supported an increase in apoptosis. There was a concentration dependent decline in the MYCN and a cyclinD1 protein indicative of anti-proliferative activity and cell cycle disruption. OXPHOS metabolism was unaffected by OZ513 treatment while glycolysis was increased. There was a significant delay in time to tumor development in mice treated with OZ513 and a decline in the rate of tumor growth.

**Conclusions:**

The antimalarial ozonide OZ513 has effective in-vitro and in-vivo activity against a pleiotropic drug resistant neuroblastoma cell-line. Treatment with OZ513 increased apoptotic markers and glycolysis with a decline in the MYCN oncogene and the cell cycle regulator cyclinD1. These effects suggest adaptation to cellular stress by mechanism which remain unclear.

## Background

Neuroblastoma is a rare childhood tumor with about 700 new cases per year in North America [1]. It is a biologically diverse tumor with clinical course and prognosis dependent on age at diagnosis, histology, and molecular pathway characteristics. A number of attempts have been made to target pathways and expression factors in neuroblastoma including mutated ALK and GD2 expression with modest success. ALK is amplified in about 14 % of neuroblastomas and while responses occur, particularly in familial cases, resistance in most sporadic cases is high and the value of the ALK inhibitor crizitonib is reduced [2]. Dinutuximab which targets GD2 gangliosides improves survival in high risk neuroblastoma when used upfront after induction and combined with GMCSF, IL-2 and isotretinoin [3]. Toxicities are substantial with this combination due to a more general expression of the GD2 antigen on normal cells and the use of IL-2. Our group has recently demonstrated the value of inhibiting sonic hedgehog pathways using vismodegib and topotecan in neuroblastoma in-vitro and in-vivo [4]. While these new therapies are promising advances in the treatment of high-risk neuroblastoma, more than half of high-risk patients die of therapy resistant disease. In addition, the aggressive combination chemotherapy used in high-risk neuroblastoma leads to severe toxicity [5]. Molecular and pathway targeting is incompletely successful because of redundant alternative growth signals which allow cancer cells to escape therapy and produce resistant disease. It may be better to target several critical basic biologic pathways in neuroblastoma tumor cells that are distinct from normal cells. The use of differentiating therapy with retinoic acid post autologous stem cell transplant has become standard of care and is an example of the success associated the use of an agent which likely affects several targets [6, 7]. The development of new therapies such as retinoic acid has occurred in minimal residual disease (consolidation/maintenance) since rates of complete remission in induction approach 100 % after intensive chemotherapy. Advances are likely to occur by maintaining the initial clinical complete remissions. Examples of processes that have a distinct cancer phenotype which may be modified to inhibit tumor growth, particularly in minimal residual disease, include cellular metabolism, autophagy, DNA repair and cell cycle regulation [8].

A basic biologic characteristic of many cancer cells is the reliance on oxidative glycolysis or the Warburg Effect (WE) which results from switching from mitochondrial based metabolism to glycolysis [8]. WE is linked to either a loss of mitochondrial mass when cells are undergoing a specialized form of autophagy called mitophagy or intrinsic abnormalities in cancer cell mitochondria resulting in a switch from mitochondrial based metabolism to glycolysis [8, 9]. This abnormal metabolism occurs not only in the cancer cells but also in microenvironmental cells, particularly cancer associated fibroblasts [10].

MYCN, an oncogene and transcription factor, amplified in neuroblastoma cells is associated with neuroblastoma growth and progression possibly by initiating both metabolic privilege mediated by WE and a high proliferative rate [11–13]. The activity of inhibitors of MYCN in high-risk neuroblastoma may partly result from inhibition of mitochondrial based metabolism [14]. Artemisinin and its analogs are natural product based therapies for malaria and include the ozonide class of antimalarials. There has been increasing interest in their anti-tumor activity including in neuroblastoma [15]. The mechanisms by which the artemisinins kill tumor remains unclear but may result from disruption of metabolism or cell cycle progression and likely via apoptosis rather than other forms of cell death [16].

The peroxide bond containing artemisinin and ozonide antimalarials may be novel treatments for chemoresistant tumors given that they are poor substrates for mdr-1 the efflux protein prevalent in cancer cells that have acquired pleiotropic drug resistance [15, 16]. In addition, these drugs have an excellent safety profile which may allow their addition to existing therapies if synergy can be shown.

In the following study we investigated the antitumor effect of ozonide antimalarials in a chemoresistant neuroblastoma cell line, BE (2)-c. Our hypothesis was that ozonides would have antitumor effects on BE (2)-c cells resulting from disruption of metabolism and cell cycle progression. Using an in-vitro assay, we identified OZ513 as the most active compound among a limited set of antimalarial ozonides. The mechanism of OZ513 was not related to inhibition of mitochondrial based oxidative phosphorylation but appear to be associated with an increase in glycolysis. There was also an increase in apoptosis potentially by modulation of cell transit in the G2/M phase of the cell cycle. OZ513 also had in-vivo activity in a pilot mouse study where OZ513 caused a significant delay in the development and rate of tumor growth in a chemoresistant minimal residual disease model.

## Methods

### Cell lines

BE (2)-c, an MYCN amplified neuroblastoma cell line (ATCC: CRL-2268) which is used to model high-risk chemoresistant neuroblastoma was used to evaluate cytotoxicity of ozonide antimalarials and investigate potential mechanism (s) of action. A 1:1 mixture of EMEM and F12 medium along with 10 % FBS was used to grow BE (2)-c and IMR-32 cells. Cells grew as adherent monolayers and were passaged using 0.25 % trypsin and 0.53 mM EDTA. Cells were passaged at a 1:4 ratio and media renewed every 3 days. All experiments were performed using cells that were 70–80 % confluent. The activity of the ozonide antimalarials were confirmed in a non-neuroblastoma cell line in addition to the two neuroblastoma cells line, type I Ewing’s Sarcoma (A673; ATCC: CRL-1598). Ewing’s A673 were plated at 1.25 × 10^5^ in a T75 flask containing DMEM, 10 % FBS, and 1 % Pen-Strep. Cells were incubated at 37 °C with 5 % CO_2_ for 7 days. 5 mL growth media was added every 2–3 days before passaging. Experiments were performed using cells that were 70–80 % confluent.

### 3-(4,5-Dimethylthiazol-2yl)-2,5-Diphenyltetrazolium (MTT) cytotoxicity assay

BE (2)-c, IMR-32, and EWS A673 cells were seeded at a density of 25,000 to 40,000 cells per well of a 96 well plate and incubated for 24 h allowing the cells to become adherent. In BE (2)-c cells chemo-resistance was confirmed by adding etoposide (25 mcg/ml), topotecan (1 μM = 458 ng/ml), cisplatin (5 mcg/ml), carboplatin (10 mcg/ml), and doxorubicin (1 mcg/ml) were studied at peak concentrations achievable in patients, and delivered in 0.01 % DMSO plus growth media. All treatments in the cancer chemotherapy screening experiments used an 18 h incubation. Cells in ozonide screening experiments were treated with a series of 12 different ozonides as well as artemisinin (ART), dihydroartemisinin (DHA), and artesunate (AS) at concentrations of 250 ng/ml, 500 ng/ml, 1 mcg/ml, 5 mcg/ml, and 10 mcg/ml for 18 h. All compounds were diluted in 0.01 % DMSO in media. Each 96 well plate included media only controls and 0.01 % DMSO plus media controls. Ten microliters of MTT (5 mg/ml) solution was added to each well and after 4 h of incubation at 37 °C DMSO was used to solubilize each well and the dark blue formazan crystals dissolved and absorption measured at 550 nm. The average absorbance of DMSO plus media controls was used to calculate a percentage of no treatment controls which was regressed against the concentration of the ozonides. This allowed the calculation of IC50 for each of the compounds tested. From these screening experiments OZ513 was determined to be the most active and was used in subsequent experiments.

### Flow cytometry propidium iodide: cell cycle analysis/apoptosis

Because ART and its analogs have been reported to disrupt cell cycle progression and increase apoptosis, varying concentrations of OZ513 were studied for analysis of effects on cell cycle progression using propidium iodide labeling and flow cytometry. Briefly, 5 × 10^5^ cells were fixed in ice cold 100 % ethanol and stored at 4 °C and analyzed within 4 weeks. Cells were washed twice and resuspended in 200 μl of propidium idodide + RNAase, incubated at 37 °C for 20–30 min, and placed on ice until analyzed by flow cytometry. Apoptosis was estimated by analysis of the Ao peak.

### Metabolic profiles associated with OZ513 treatment

A mitochondria stress test and glycolysis stress test with and without OZ513 treatment were performed using a Seahorse® metabolic analyzer which measures OXPHOS metabolism as measured by oxygen consumption rate (OCR) and glycolysis as measured by extracellular acidification rate (ECAR). Analysis was performed with and without an 18 h pre-treatment with OZ513.

### MYCN, cleaved capase-3, CyclinD1, and cleaved PARP western blots

MYCN, capase-3, Cyclin D1, and PARP protein was measured with and without OZ513 treatment at varying concentrations of 0.5, 1, 2.5, and 5.0 mcg/ml for 18 h. Briefly, total proteins were isolated from BE (2)-c cells using RIPA lysis buffer and protein quantified using the BCA assay. Protein was loaded (20 mcg) and resolved on precast polyacrylamide gels and transferred onto nitrocellulose membranes. The primary antibody for MYCN, cleaved capase-3, Cyclin D1, and cleaved PARP were used at a dilution of 1:1000 per manufacturer’s recommendations. Beta-actin or GAPDH served as a loading control. A rabbit anti-mouse IgG secondary antibody was used at a dilution of 1:2000. Detection was performed using a MyECL Imager (ThermoScientific, MA, USA) and band density was normalized using the measurement of total protein.

### Growth of BE (2)-c in NSG Mice with and without OZ513 Treatment

The use of NSG mice to test the activity of OZ513 was approved by UNMC IACUC (protocol#: 13-050-00-Fc). NSG mice (*N* = 12) were injected subcutaneously with 1 × 10^6^ BE (2)-c cells in a 50:50 PBS/Matrigel® solution. Starting on the date of tumor implantation mice began 3 times per week injections of OZ513 at a dose of 100 mg/kg per injection. After the first three loading doses, the dose was lowered to 50 mg/kg for the remainder of the study out to day 23.

### Statistical analysis

Time to tumor development was determined using Kaplan-Meier analysis and differences between time to tumor development curves in treated and control mice were determined using the log-rank test. Comparison testing for multiple groups was performed using Kruskall Wallis and Wilcoxon matched-pairs sign ranked test. Statistical significance was defined as *p* ≤ 0.05.

## Results

### Cytotoxicity screening of 12 ozonides, artemisinins, and cytotoxic chemotherapy

**Fig. 1:**
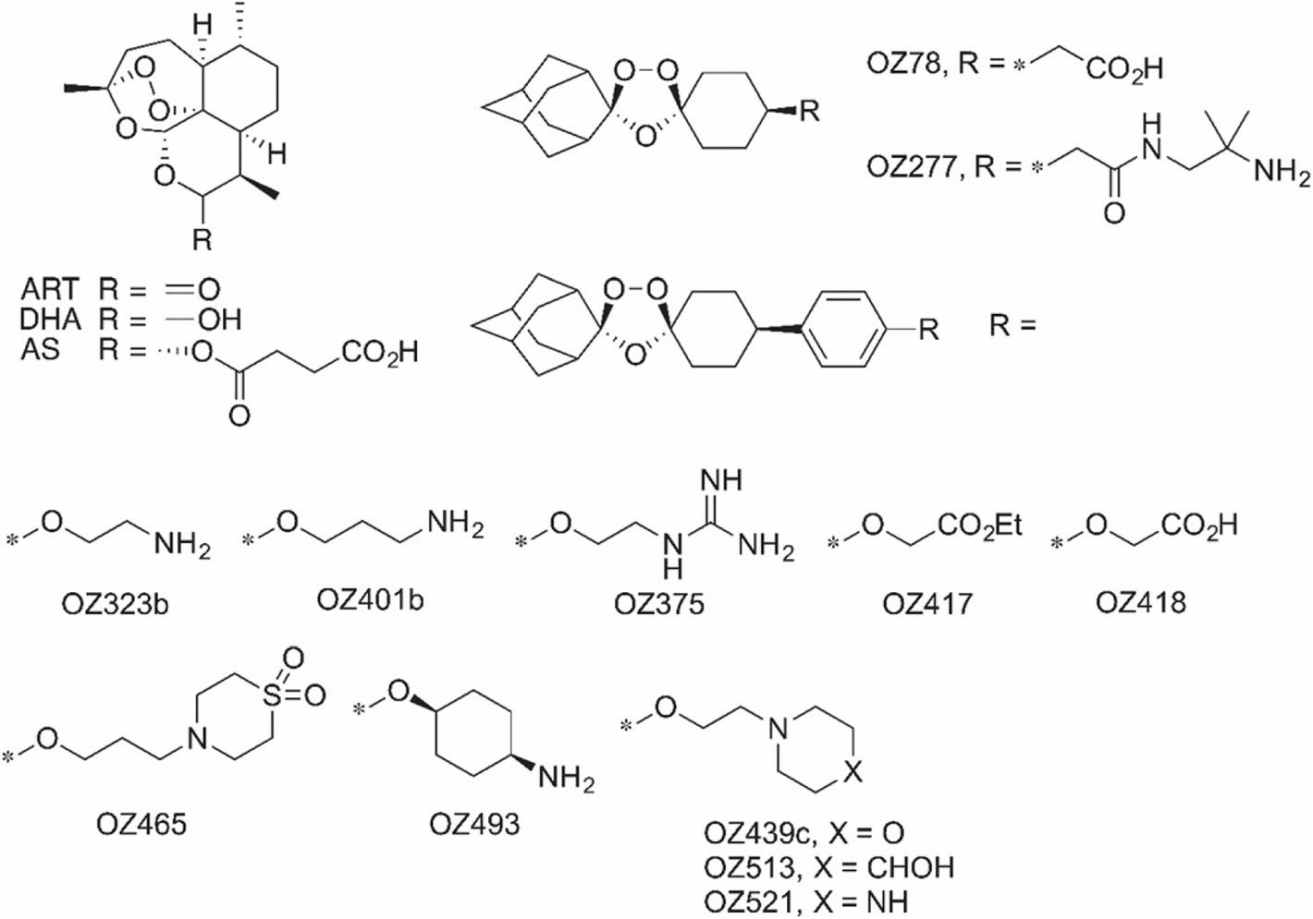
Chemical structures of Ozonide Antimalarials and parent compounds artusunate (AS), artemisinsin (ART), and dihydroartemisinsin (DHA)

**Fig. 2:**
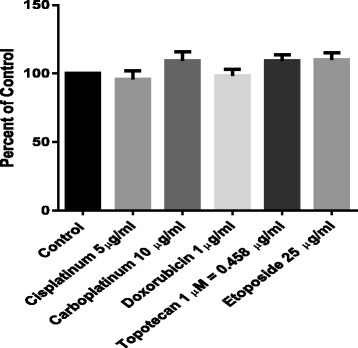
Activity of Chemotherapy Drugs used in high-risk neuroblastoma. Graphed as percentage of no treatment control in BE (2)-c neuroblastoma cells. Concentrations studied were those achievable in patients

Figure 1 gives the chemical structures of 12 ozonide antimalarials along with the artemisinin analogs ART, DHA, and AS. ART, DHA, and AS were selected for study based on their structural relationship to the ozonides and their early development as antimalarials and potential treatments for cancer [16]. Figure 2 illustrates the high level resistance of the BE (2)-c to etoposide, topotecan, doxorubicin, cisplatin, and carboplatin all drugs commonly used in the treatment of neuroblastoma. The concentrations of chemotherapy used were those that could be obtained in patients at peak concentrations. The high concentrations used for etoposide (25 mcg/ml) were those that can be achieved after high dose therapy in the setting of autologous stem cell transplants [17]. These experiments confirmed the high level of chemoresistance of BE (2)-c cells. These data confirmed that this cell line served as a model for chemoresistant neuroblastoma for the evaluation of ozonide compounds as therapy in refractory neuroblastoma.

**Fig. 3:**
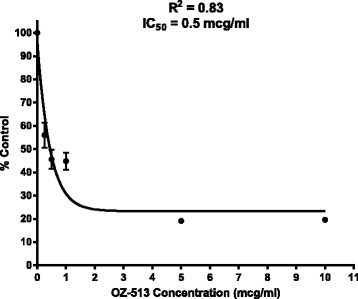
Concentration versus response of OZ513 in BE (2)-c cell culture using MTT viability assay. Concentrations of OZ513 studied were 0, 0.25, 0.5, 1, 5, and 10 mcg/ml. Activity was measured as a percentage of DMSO controls (0.01 % DMSO in growth media). All drug concentrations were diluted in 0.01 % DMSO in growth media identical to DMSO controls

Table 1 lists the ozonide compounds and IC50 values in BE (2)-c, IMR-32, and A673 cell culture. While a number of compounds had single digit microgram/ml IC50’s, OZ513 was the most active with an IC50 of 0.5 mcg/ml in BE (2)-c. Interestingly, OZ513 was approximately six-fold less active against the EWS-A673 cell line and IMR-32 cell line. The higher IC50’s in EWS-A673 and IMR-32 cell lines were also seen with the other ozonide compounds active against BE (2)-c. Because OZ513 had the highest activity in the BE (2)-c, the cell line used to model chemoresistant neuroblastoma, it was selected as the representative ozonide in the subsequent experiments. A time course of OZ513 effect was performed to determine time to optimal effect and 18 h was used for subsequent experiments corresponding to an overnight incubation period. Concentration versus response curve for OZ513 is shown in Fig. 3.

**Table 1:** Concentration versus activity of ozonide antimalarials and artemisinin in BE (2)-c and IMR-32 neuroblastoma and Ewing’s Sarcoma. IC50 calculated from concentration versus absorbance (response) graph with concentrations of 0, 0.5, 1, 2.5, 5, and 10 mcg/ml. Ten measurements were obtained at each concentration. All drugs and control were in 0.01 % DMSO and growth media

	Compound	IC50 (mcg/ml) BE (2)-c	IC50 (mcg/ml) IMR-32	IC50 (mcg/ml) EWS A673
1	OZ323	6.0	6.1	6.2
2	OZ375	5.8	3.9	>10
3	OZ418	>10	>10	>10
4	OZ401	>10	>10	>10
5	OZ465	>10	>10	>10
6	OZ78	>10	4.5	>10
7	OZ439	>10	5.6	>10
8	OZ277	>10	>10	>10
9	OZ521	1.4	>10	5.8
10	OZ493	2.2	>10	6.9
11	OZ417	>10	3.4	>10
12	OZ513	0.5	3.1	3.3
13	DHA	3.2	>10	NT
14	ART	>10	NT	NT
15	AS	>10	NT	NT

### Metabolic effect of OZ513 on BE (2)-c neuroblastoma cells

**Fig. 4:**
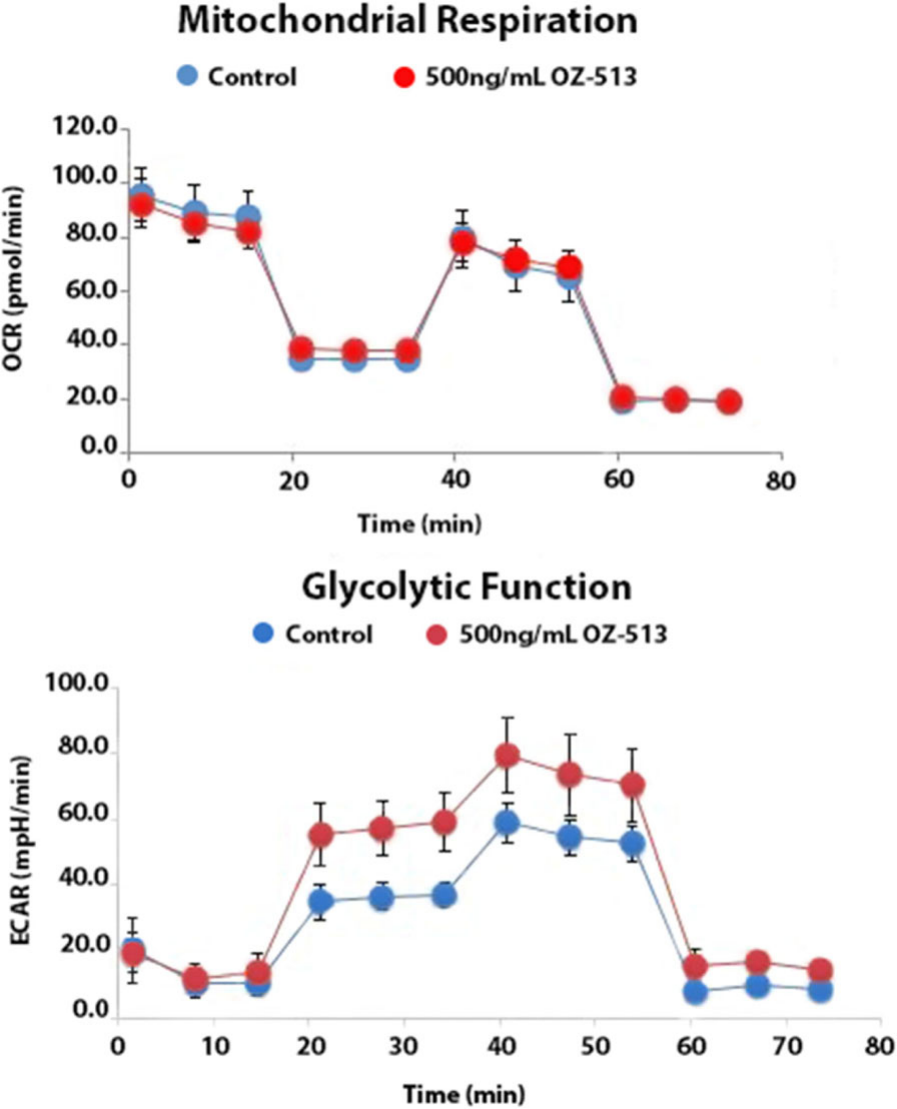
Metabolic profile as measured by oxygen consumption rate (OCR) and extracellular acidification rate (ECAR). OZ513 studied after an 18 h pre-treatment at a concentration of 500 ng/ml. Control was media alone and experimental group was treatment with OZ513

OZ513 at the IC50 concentration of 0.5 mcg/ml had no effect on OXPHOS. There was a more robust response in OZ513 treated cells after the injection of glucose when compared to non-treated controls. This may suggest a compensatory increase in glycolysis as a potential stress response after treatment with OZ513 (Fig. 4).

### Cell cycle analysis

**Fig. 5:**
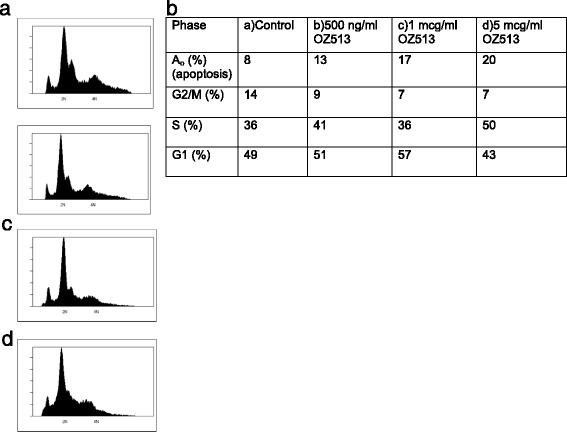
Propidium iodide labeled flow cytometry for cell cycle analysis in BE (2)-c cells after 18 h treatment with 0, 0.5, 1 and 5 mcg/ml of OZ513. Varying concentrations of OZ513 were added to BE (2)-c cell culture for cell cycle analysis: (**a**) 0, (**b**) 500 ng/ml, (**c**) 1 mcg/ml and (**d**) 5 mcg/ml). Results show a concentration dependent increase in the percentage of live cells undergoing apoptosis indicated by increasing Ao peak with increasing concentrations of OZ513

Cell cycle analysis showed a concentration dependent increase in apoptotic Ao peak on flow cytometry indicative of increased apoptosis. In addition there was a reduction in cells transiting G2/M in treated versus non-treated cells. There was also a suggestion of increased S-phase fraction at the highest concentration of OZ513 tested (5 mcg/ml). Cell cycle histograms are shown in Fig. 5.

### MYCN, CyclinD1, cleaved capase-3, and cleaved PARP western blot

**Fig. 6:**
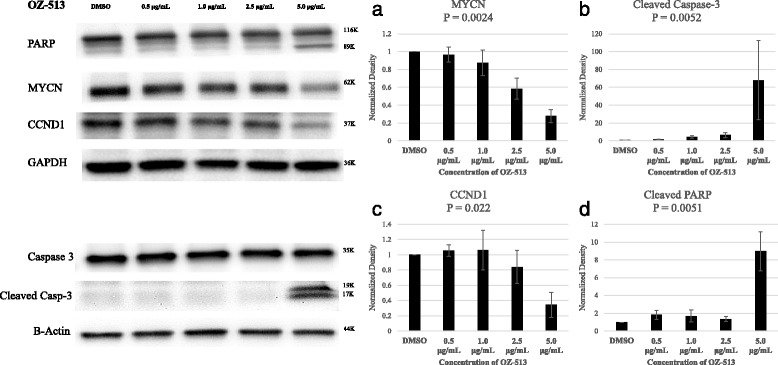
**a** MYCN, **b** capase-3 and cleaved capase-3, **c** CyclinD1, and **d** PARP and cleaved PARP protein after treatment with 0.5, 1, and 2.5 mcg/ml OZ513. Treatment and control diluted in 0.01 % DMSO in growth media

There was a statistically significant concentration dependent decline in MYCN protein after treatment with OZ513 (Fig. 6a). In support of the cell cycle analysis which showed a concentration dependent increase in Ao peak on flow cytometry indicative of increased apoptosis there was also a statistically significant concentration dependent increase in cleaved capase-3 and cleaved PARP proteins by western blot (Fig. 6 b, d). There was also an increase in S-phase fraction on cell cycle analysis in cells treated with 5 mcg/ml corresponding to a significant decline in CyclinD1 on western blot (Fig. 6c).

### OZ513 treatment effect on tumor growth in NSG mice

**Fig. 7:**
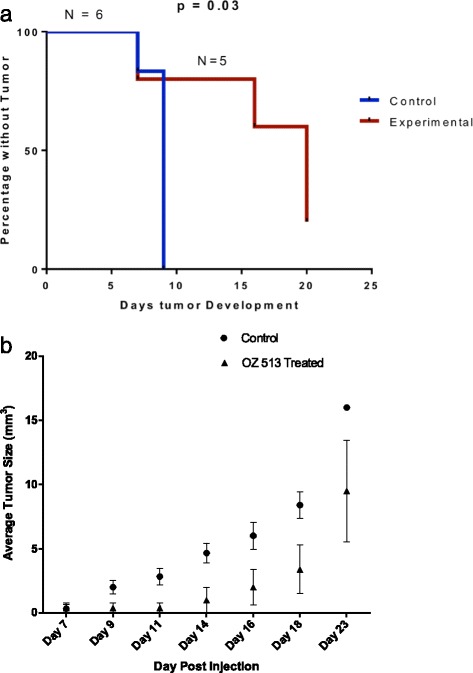
**a** Time to development and incidence of BE (2)-c tumors after injection of 1 × 10^6^ BE (2)-c cells subcutaneously (**b**) Average tumor development and growth rate

OZ513 treatment led to a significant delay in tumor development. All six control mice developed tumors by day 9 after subcutaneous injection of BE (2)-c cells (1 × 10^6^ cells). In treated mice one mouse died prematurely of causes unrelated to drug or tumor and was excluded from the analysis leaving 5 treated mice. Median time to tumor development was day 19 and one of the five treated mice did not develop tumor (Fig. 7a). There was a statistically significant lower incidence of tumor development and time to tumor development in the treatment group (*p* = 0.03). Median time to tumor development was day 9 for no treatment controls versus day 18 for the OZ513 treated group. Average tumor growth rate is included as Fig. 7b.

## Discussion

Neuroblastoma is the most common extra cranial solid tumor occurring in children, and the treatment of metastatic disease continues to be challenging. Especially problematic is the treatment of children with high-risk disease, who have survival rates of less than 40 % at 5 years, despite aggressive multimodal treatment [18]. Therapy failures are from relapsed chemoresistant disease. Therefore, innovative approaches to the treatment of neuroblastoma are needed.

Ozonide antimalarials are synthetic peroxide mimics of artemisinin, a sesquiterpene lactone endoperoxide natural product discovered from traditional Chinese medicine. Ozonide OZ277 (arterolane) is marketed in combination with piperaquine to treat uncomplicated malaria [19]. OZ439 is currently undergoing development as an anti-malarial, also in combination with piperaquine [20]. The proposed mechanism of action in malaria relates to the alkylation of heme and parasite proteins after reductive activation by ferrous iron in the food vacuole of the malarial parasite [21]. Indeed, the extent of heme alkylation correlates to ozonide antimalarial potency [22]. In addition, weak base and neutral ozonides are more active against malarial parasites compared to their weak acid counterparts [23]. As noted by the differences in IC50’s in Table 1, the targets for the ozonides in the treatment of cancer is likely different from those in malaria parasites. For example, OZ277 and OZ439 are highly active against the malarial parasite but have no significant activity against chemoresistant neuroblastoma or Ewing’s Sarcoma cell lines. However, similar to the antimalarial structure-activity-relationship (SAR), weak base ozonides OZ323, OZ521, OZ493, and OZ513 were more potent than weak acid ozonides OZ418 and OZ78 in chemoresistant neuroblastoma and Ewing’s Sarcoma. Activity differences seen between BE (2)-c and IMR-32 are substantial and these differences will form the basis for future mechanism studies. One potential explanation is that OZ513 inhibits MYCN which is highly amplified in BE (2)-c cells and intermediately amplified in IMR-32 [24]. Other mechanisms are likely involved and more extensive structure activity relationships will be required after screening a larger library of ozonides. The activity data in Table 1 does support that OZ513 is the most active of the ozonides studied in all three cell lines.

The semisynthetic artemisinins, and more recently, ozonide OZ439 have been studied as potential anti-cancer agents [25, 26]. There are a number of proposed mechanisms to account for the activity of artemisinin and its analogs in cancer. Extending mechanism studies from malaria to cancer, the role of ferrous iron and alkylation of heme has been proposed given the increased synthesis of heme in cancer cells as well as increased requirements of iron in many cancers [27]. This hypothesis has been refined to a proposed interaction of heme associated with cytochrome c in the mitochondria, with the production of reactive oxygen species, and induction of apoptosis.

Mitochondria of cancer cells have many differences when compared to those from normal cells and ozonide-mediated generation of ROS in the mitochondria may be an important mechanism of anti-cancer action. In general, mitochondria in cancer are more negatively charged than normal mitochondria and the positively charged weak base ozonides may differentially accumulate in cancer mitochondria [28]. Data presented here indicates that any effect of OZ513 on mitochondrial function does not appear to result from the uncoupling of OXPHOS metabolism given the lack of effect on oxygen consumption rate. While we report a dose-dependent increase in apoptosis after treatment with OZ513 the lack of effect on OXPHOS metabolism would suggest apoptosis is occurring by extrinsic rather than intrinsic pathways [29, 30]. The modest increase in lactic acid production in OZ513 treated cells would suggest the cells are metabolically stressed which increases oxidative glycolysis.

The single agent activity of OZ513 in a chemoresistent neuroblastoma cell line is impressive. BE (2)-c is highly MYCN amplified and has pleotropic drug resistance likely as a result of upregulation of multiple resistance mechanisms but in particular the efflux proteins MDR-1 [24]. It is of interest that other investigators have reported that artemisinin and its analogs are not substrates for MDR-1 [31].

While no data is available on OZ513 toxicity, class toxicity for the ozonides is fairly well described in the rat. Five doses as high as 300 mg/kg have been administered orally every three days and toxicity based on clinical observation, body weight changes, clinical laboratories (hematology, chemistries, and urinalysis), and necropsies including organ histology was low [20]. There were no significant changes in body weight or blood and urine parameters with only minor gastric irritation which was reversible. This excellent toxicity profile was confirmed in the first in man safety study including in children [32, 33].

While the anticancer activity of the ozonides studied in these experiments is largely restricted to weak bases, not all of the weak bases tested had potent anticancer activity. In fact, OZ439 had very low anticancer activity in BE (2)-c cells (IC25 = 9.6 mcg/ml). Apparently a weak base functional group may be required but is not sufficient for anticancer activity. The fact that OZ439 and other active antimalarials were not potent anticancer agents might suggest different targets in malaria compared to cancer.

## Conclusion

This new class of anticancer agents showed promising in-vitro and in-vivo activity in a highly resistant neuroblastoma cell line. Future studies centered on mechanism of action will allow a rational experimental therapeutic approach where combinations are prioritized based on complimentary targets. The good safety profile of ozonides in malaria, even at high doses, will be advantageous in integrating these compounds into treatment regimens for neuroblastoma where minimization of the severe effects of treatment related toxicities in the pediatric population is increasingly important.
